# A Probable Fatal Case of Oleander (*Nerium oleander*) Poisoning on a Cattle Farm: A New Method of Detection and Quantification of the Oleandrin Toxin in Rumen

**DOI:** 10.3390/toxins11080442

**Published:** 2019-07-25

**Authors:** Silva Rubini, Sabina Strano Rossi, Serena Mestria, Sara Odoardi, Sara Chendi, Andrea Poli, Giuseppe Merialdi, Giuseppina Andreoli, Paolo Frisoni, Rosa Maria Gaudio, Anna Baldisserotto, Piergiacomo Buso, Stefano Manfredini, Guido Govoni, Stefania Barbieri, Cinzia Centelleghe, Giorgia Corazzola, Sandro Mazzariol, Carlo Alessandro Locatelli

**Affiliations:** 1 Experimental Institute for Zooprophylaxis in Lombardy and Emilia Romagna, 25100 Brescia, Italy; 2 Institute of Legal Medicine, Catholic University of Sacred Heart, 00168 Roma, Italy; 3 Azienda USL di Ferrara Unità Operativa Attività veterinarie, Distretto Sud-Est Ufficio di Comacchio, 44022 Comacchio (FE), Italy; 4 Department of Medical Science, University of Ferrara, 44121 Ferrara, Italy; 5 Department of Life Science and Biotechnology, University of Ferrara, 44121 Ferrara, Italy; 6 International Committee of the Red Cross—ICRC, 1202 Genève, Switzerland; 7 Department Urgency University of Padova, 35122 Padova, Italy; 8 Department of Biomedicine and Food Science BCA, University of Padova, 35020 Legnaro (PD), Italy; 9 Pavia Poison Control Centre—National Toxicology Information Centre, ICS Maugeri Hospital, 27100 Pavia, Italy

**Keywords:** oleander, *Nerium oleander*, poisonous plant, cattle, LC–HRMS

## Abstract

Oleander (*Nerium oleander*) is an ornamental plant common in tropical and sub-tropical regions that is becoming increasingly widespread, even in temperate regions. Oleander poisoning may occur in animals and humans. The main active components contained in the plant are cardiac glycosides belonging to the class of cardenolides that are toxic to many species, from human to insects. This work describes a case of oleander poisoning that occurred on a small cattle farm and resulted in the fatality of all six resident animals. Furthermore, the investigation of the poisonous agent is described, with particular focus on the characterization of the oleandrin toxin that was recovered from the forage and rumen contents. The innovation of this study is the first description of the detection and quantification of the oleandrin toxin by liquid chromatography-high resolution mass spectrometry (LC-HRMS) in rumen.

## 1. Introduction

Common oleander (*Nerium oleander*) is a plant belonging to the Apocynaceae family, which can reach a height of 2–6 metres. This species is native to the Mediterranean regions of Europe and Africa [1]. Oleander is widely distributed in tropical and sub-tropical regions but is also widespread in temperate areas. In many countries, oleander is cultivated as an ornamental plant due to its beautiful flowers, and due to its resistance to many plant diseases it is also used as landscaping along roadsides. Another favourable characteristic of this plant is its adaptability to various types of soils without requiring special treatments [1,2,3].

Non-digitalis cardiac glycosides, contained throughout the plant, are among the most poisonous compounds found in plants and have toxic and sometimes fatal side effects, including gastric disorders (vomiting, nausea and burning), bradycardia, and increased respiratory rate and central nervous system disorders, including lethargy.

Cardiac glycosides are a family of molecules that are potent inhibitors of the Na^+^/K^+^-ATPase and induce electrolytic disturbances that affect the electrical conductivity in the heart and in other body regions, which can have toxic results. Cardioactive glycosides are divided into two different main components: (i) A carbohydrate moiety that may be a single component or a poly-glycoside, which is important for solubility and influences absorption and biodistribution; (ii) an aglyconic portion that is divided into a steroid backbone characterized by a typical “U” structure due to the specific CIS–TRANS–CIS fusion in the ring and an attached unsaturated lactone ring at the C-17 position. This latter moiety has a role of primary importance in the interaction with its receptor, probably for the carbonyl oxygen interaction with the binding site of the Na^+^-K^+^ ATPase pump and other side interactions. Indeed, the unsaturated lactone ring at position 17 and the hydroxyl group at position 14 of oleandrin have a direct influence on the inhibitory activity of Na^+^ K^+^-ATPase.

**Figure 1:**
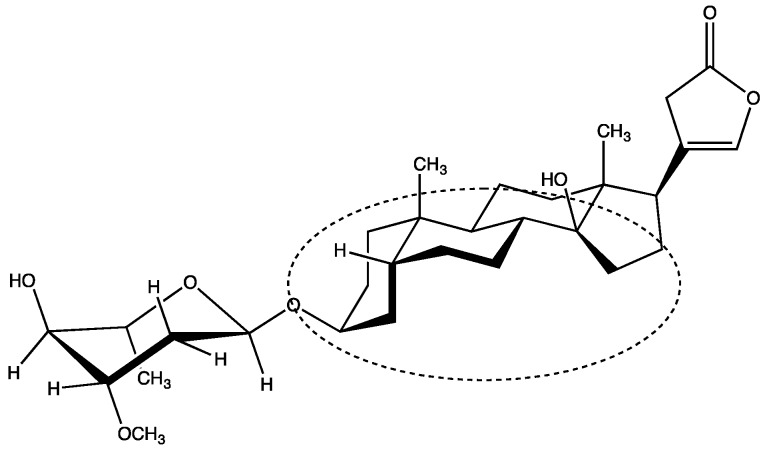
Structure of oleandrin with the characteristic U-shaped structure circled.

Moreover, these compounds can be categorized based on the source from which they were discovered: Cardenolides (*Digitalis purpurea* and *lanata*), which are contained in plant tissues, and bufadienolides, which were first discovered as skin poisons produced by glands of certain species of toads (*Bufo marinus*) and by some species of plants [4,5,6]. Together with the alkaloids, oleandrin (Figure 1), a cardiotoxic glycoside (structurally similar to ouabain), which inhibits the sodium-potassium pump by binding at the ouabain site of the cell membrane, is responsible for this extreme toxicity. However, oleander contains a number of other toxic components, which are preserved even after drying.

Other natural substances with the same mechanism of action include digoxin and digital purpurea. *N. oleander* contains more than 30 different toxic cardiac glycosides belonging to the class of cardenolides, with the most important detected compounds being oleandrin, oleandrigenin, digitoxigenin, neriin, folinerin, and rosagenin [3,7,8,9].

Many cardenolides [10] are present in different parts of the plant. In all cases where the toxin is detectable, its concentration is sufficient to cause significant clinical manifestations.

The percentage of cardenolides within the plant substructures follows this descending order: seeds = roots > fruits > leaves. The total content of cardenolides is indicatively recognizable by the colour of the flowers; the content of cardenolides is greater in plants with red flowers than in those with white flowers [1].

**Figure 2:**
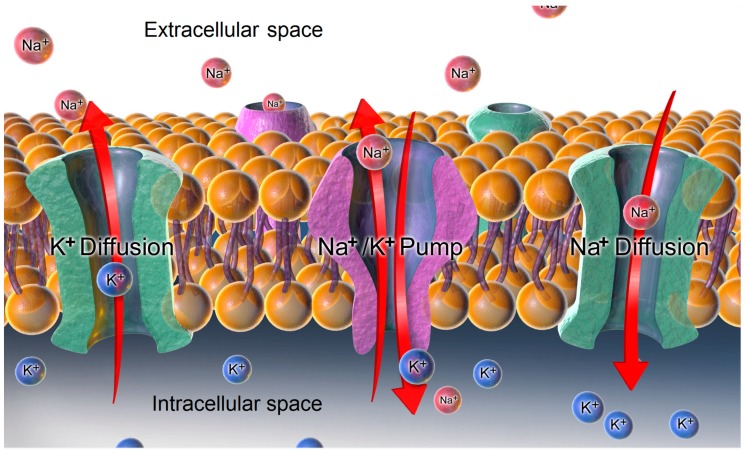
Representation of the Na⁺/K⁺-ATPase Pump. Blausen.com staff (2014). Reproduced from [15]. This file is licensed under the Creative Commons Attribution 3.0 Unported license 2014, Wiki Journal of Medicine.

**Figure 3:**
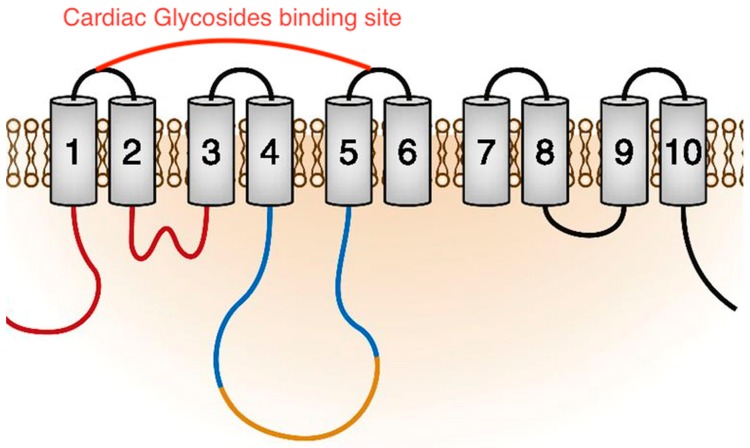
Schematic representation of the Na^+^-K^+^-ATPase pump trans-membrane domains with the extracellular Ouabain binding site indicated.

As reported above, due to their similarity with digitalis glycosides, the cardenolides inhibit the Na^+^-K^+^-ATPase pump in myocytes (Figure 2). The binding occurs within an extracellular portion of the pump, with *N. oleander* cardenolides inducing a conformational change in the enzyme by stabilizing it in the transition state, which decreases the active transport of sodium with the consequent excretion of potassium (Figure 3). Increased intracellular sodium levels lead to the inhibition of NCX (Na^+^/Ca^2+^ exchanger) and, thus, to an increase in intracellular levels of Ca^2+^ that are responsible for the cardenolides’ positive inotropic effect and toxicity [4,11,12,13,14].

In particular, the most important cardiac glycoside in this plant is oleandrin, due to its prominent lipophilicity (only two hydroxy groups in the whole structure) that results in a slow urinary excretion rate, rapid and conspicuous gastrointestinal absorption, with a bioavailability of approximately 30% [16], and longer duration of action as a cardiotoxic agent. Despite constituting approximately 0.08% of the total amount of cardenolides in the plant, oleandrin is the main component responsible for oleander toxicity because of the above described characteristics [8].

Oleander is considered the most important cause of livestock poisoning in South Africa [17]. Accidental intoxications have been reported in horses [18,19,20,21], donkeys [22], cattle [23,24,25], camelids such as llama and alpaca [26,27], dogs [9,11,28], cats [11,29], and pet birds [30].

Although oleander is unpalatable to animals, in some circumstances animals can swallow it [11]. This ingestion can occur, for example, in pets that are bored and curious but also hungry [3]. In arid areas or in prolonged drought periods, animals may spontaneously swallow oleander leaves or seeds, but poisoning usually occurs by ingestion of different parts of the plant, for example, pruning residues that are accidentally blended with forage. As described above, oleander is toxic even for humans, particularly for children [31]. In children, a single leaf may be lethal [32]. While poisoning in animals is typically due to accidental circumstances, in humans, poisoning episodes can be the result of voluntary acts, including suicide [33,34,35,36,37,38] and malicious or criminal cases [39,40].

Additionally, in South Asia, cardenolides from pink, white or yellow oleander are a leading cause of poisoning, including for suicide, with thousands of cases a year and a fatality rate from 5% to 10% [41]. Accidental poisoning also occurs by inhaling toxic fumes, due to the use of parts of the plant as firewood for a barbecue [42]. When the quantity of toxic substances is not immediately lethal, colic pains, weakness, ruminal atony, hypersalivation, changes in normal heart rhythm, dyspnea and coma can be observed. In addition, renal damage has been noted in horses with oleander toxicosis [7,20,28]. Symptoms at the cardiac level are usually arrhythmias because of increased sympathetic tone, leading to sinus arrest, tachycardia, bradycardia or atrioventricular block. Mydriasis in animals, after oleander ingestion, is also observed in relation to the increased sympathetic tone [15].

The purpose of this work is not only to report a case of poisoning on a cattle farm but also to emphasize the hazard of oleander ingestion for humans and animals and, thus, the need for effective methods of detection. In fact, although the toxic properties of this plant have been well known for a long time, reports of poisoning cases in humans and animals still recur in the scientific literature.

Moreover, during the investigation of this poisoning case, a new method for the detection and quantification of oleandrin toxin in rumen and forage has been developed. A liquid chromatography (LC) system coupled with high resolution mass spectrometer (HRMS) was used after the extraction of the studied samples [43].

## 2. Results and Discussion

Fatal oleandrin intoxications are still a matter of concern for public health, despite its well-known toxicity.

The aim of this paper is to provide a reliable method for the determination of oleandrin, performed by liquid chromatography (LC) high resolution mass spectrometry (HRMS) after a simple extraction from biological samples, such as cattle feed.

First, a macroscopic examination of the studied samples was conducted, in which oleander leaves were found in all samples. Upon examination of the forage sample, 50.4 g of oleander leaves was isolated from a total weight of 1922 g.

Establishing the lethal dose is rather difficult both in cattle and in other animal species because the evaluation is affected by many variables, depending on the quantity and characteristics of the ingested vegetal material and on the organism undergoing the poisoning. Specifically, it is necessary to consider the following: The parts of the plant that have been ingested, the quantity of plant ingested, the physiological state of the animal at the moment of ingestion, and age and current state of health of the subject.

According to Oryan et al. [44], the minimum oral lethal dose (LD) in cattle is 50 mg/kg of oleander leaves. Other authors report a LD of green or dried leaves of 110 mg/kg [45]. According to other researchers, a number of oleander leaves between three and ten can be considered lethal to a bovine adult [23]. The amount of toxic material found in the forage samples far exceeds the LD indicated in the literature.

The chemical examination performed through LC-HRMS detected 2700 ng/mg in the leaves taken from the forage, 320 ng/mL in the ruminal liquid content and 294 ng/g in the solid rumen content.

The delayed reporting by the farmer to the veterinary service did not allow a rapid execution of an accurate necropsy on the deceased animals which, at the time of the veterinary intervention, were already in an incipient state of decomposition. Therefore, it was not possible to perform a necropsy and to obtain blood samples from the carcasses. Despite the lack of necropsy examinations and laboratory investigations on blood samples obtained from the animals involved, the macroscopic examination of the rumen content associated with anamnestic surveys allowed us to detect and quantify the presence of parts of the oleander plant and to express a concrete diagnostic judgement of oleander poisoning, similar to other cases already described and reported in the literature [23]. Subsequent laboratory investigations of forage and rumen content samples described in this paper have unequivocally confirmed the diagnosis of poisoning caused by oleander plant parts. Taken together, these data likely indicate oleander ingestion as the cause of death. However, the late reporting by the farmer that prevented the blood analysis and the lack of recovery of two of the six dead animals does not allow the cause of death to be attributed with absolute certainty and leaves a margin of uncertainty.

## 3. Materials and Methods

### 3.1. Case Presentation

The poisoning episode involved a small cow-calf operation cattle farm. This farm employed free-range farming for breeding its cattle; in fact, the farmer used a vast plot of land where abundant vegetation was present. There were a total of 6 cattle on the farm. The breeder found 4 dead animals in the barn on Saturday, and the remaining 2 animals were never found. The following Monday morning, the breeder informed the Official Veterinary Service, which intervened a few hours after the report. The four cattle were found at the corners of the barn. The anamnestic investigation found that the animals had been fed pruning residues from a section of land in which oleander was present.

Three specimens were received at the laboratory: Two specimens from the rumen content of two different cattle (one liquid and the other solid), and one specimen from a forage sample.

The three samples were subjected to macroscopic and microscopic examinations. The leaf fragments found in the rumen contents were small and not easily isolatable from the rest of the contents. However, there were leaves in the forage sample that were clearly distinguishable by the direct examination. The entire forage sample was first weighed, after which all parts of recognizable leaves were collected and weighed.

Sub-samples of the rumen contents and the leaves isolated from the forage were sent to the laboratory of the Institute of Legal Medicine, Catholic University of Sacred Heart, Roma (Italy) for the detection of oleandrin.

### 3.2. Chemicals and Reagents

Formic acid and ultrapure water were purchased from 3V-Chemicals (Rome, Italy). Ammonium formate was purchased from Sigma-Aldrich (Milan, Italy). Methanol was purchased from Merck (Darmstadt, Germany). All solvents used were LC-MS grade.

Oleandrin and testosterone-D3 were from Sigma-Aldrich (Milan, Italy). Individual methanolic solutions of standards were prepared from the pure powder at a concentration of 1 mg mL^−1^ and stored at −20 °C according to the manufacturer’s specifications. Two working solutions, each at a concentration of 1 μg mL^−1^ in methanol, were then prepared by diluting the stock solutions in methanol and stored in the freezer at −20 °C.

### 3.3. Sample Preparation

Samples were analysed in order to verify the presence of oleandrin in the liquid and solid fractions of the rumen content. Aliquots of the rumen content were collected in glass tubes and centrifuged for 5 min at 4000 rpm in order to separate the liquid from the solid contents, which included leaves swallowed but partially intact. The supernatant (100 µL) was diluted with 100 µL of internal standard working solution.

One gram of solid material obtained after centrifugation was weighed, and 5 mL of methanol and internal standard working solution (final concentration 100 ng/g) were added. One gram of oleander leaves collected from the forage were prepared in the same way. The mixtures were extracted in an ultrasonic bath for 120 min, and the extracts obtained were then diluted 1:1 with methanol and injected into the LC-HRMS system.

### 3.4. LC-HRMS

#### 3.4.1. Equipment

The LC–HRMS system was composed of a Thermo ULTIMATE 3000 system equipped with an analytical column, a Thermo Acclaim RSLC 120 C18 (2.1 mm × 100 mm, 2.2 μm particle size), coupled to a Thermo single-stage Orbitrap (Exactive) MS system. The entire system was provided by Thermo Fisher Scientific (Milan, Italy).

#### 3.4.2. LC-HRMS Conditions

Mobile phase A was ultrapure water with 0.1% formic acid and 5 mM ammonium formate, and mobile phase B was a solution of methanol with 0.1% formic acid. The analytical column was maintained at 40 °C, and the sample injection volume was 10 μL. The flow rate was set at 400 μL/min. The mobile phase gradient was as follows: 60% A for 1 min, a linear gradient to 100% B for 7 min followed by a 5 min hold, a column re-equilibration with a linear gradient to 60% A for 3 min, followed by a 3 min hold.

The HESI source was heated at 400 °C. The source current was 6 µA, the sheath gas and auxiliary gas (both nitrogen) flow rates were 35 and 5 arbitrary units, respectively, and the capillary temperature was 290 °C.

The data were acquired in full scan mode over a mass range of 110–800 *m*/*z*. The instrument was operated in positive ion mode with a resolving power of 100,000 FWHM.

Mass calibration was performed according to the guidelines provided by the instrument supplier. The recommended calibration solution was made of MRFA (L-methionyl-arginyl-phenylalanylalanine acetate), caffeine and Ultramark® 139 1621 dissolved in methanol/water (1:1). For calibration purposes, the automatic calibration feature of the Exactive Tune software was used. The mass scale was calibrated every 2 days over the mass range of 50–2000 *m*/*z*.

Lock-mass (diisooctyl phtalate ionic species, 391.2843 *m/z*) was utilized during the analysis of the samples in order to compensate for any possible mass axis drifts.

#### 3.4.3. Data Analysis

Identification was based on the following criteria: Retention time window (±0.2 min); exact mass of monoisotopic ion M+0 (mass accuracy _m, defined as (exact mass)–(accurate mass)/(accurate mass) × 106, less than 2 ppm); comparison of experimental and calculated isotopic patterns (Relative Isotopic Abundances RIA (M+1/M+0) and/or RIA (M+2/M+0) errors less than 15%).

Xcalibur 2.1 software (Thermo Fisher Scientific Inc., San Jose, CA, USA) was used to analyse and process all data for quantitative analysis [43].

## References

[1] Bandara V., Weinstein S.A., White J., Eddleston M. (2010). A review of the natural history, toxinology, diagnosis and clinical management of Nerium oleander (common oleander) and Thevetia peruviana (yellow oleander) poisoning. Toxicon.

[2] Khan I., Kant C., Sanwaria A., Meena L. (2010). Acute Cardiac Toxicity of Nerium Oleander/Indicum Poisoning (Kaner) Poisoning. Heart Views.

[3] Anadón A., Martínez-Larrañaga M.R., Castellano V., Gupta R.C. (2012). Poisonous plants of Europe. Veterinary Toxicology.

[4] Langford S.D., Boor P.J. (1996). Oleander toxicity: An examination of human and animal toxic exposures. Toxicology.

[5] Knight A.P., Knight A.P. (2003). Nerium oleander. A Guide to Poisonous House and Garden Plants.

[6] Omidi A., Razavizadeh A.T., Movassaghi A.R., Aslani M.R. (2012). Experimental oleander (Nerium oleander) intoxication in broiler chickens (Gallus gallus). Hum. Exp. Toxicol..

[7] Al B., Yarbil P., Dogan M., Kabul S., Yildırım C. (2010). A case of non-fatal oleander poisoning. BMJ Case Rep..

[8] Praveen U.S., Gowtham M.D., Yogaraje-Gowda C.V., Nayak V.G., Mohan B.M. (2012). Detection of Residues of Cardenolides of Nerium oleander by High-Performance Thin-Layer Chromatography in Autopsy Samples. Int. J. Med. Toxicol. Forensic Med..

[9] DeClementi C., Pao-Franco A., Hammond T.N., Weatherton L.K., Forney S.D. (2017). Successful use of digoxin-specific immune Fab in the treatment of severe Nerium oleander toxicosis in a dog. J. Vet. Emerg. Crit. Care.

[10] Agrawal A.A., Petschenka G., Bingham R.A., Weber M.G., Rasmann S. (2012). Toxic cardenolides: Chemical ecology and coevolution of specialized plant–herbivore interactions. New Phytol..

[11] Milewski L.M., Khan S.A. (2006). An overview of potentially life-threatening poisonous plants in dogs and cats. J. Vet. Emerg. Crit. Care.

[12] Kanji S., MacLean R.D. (2012). Cardiac Glycoside Toxicity More Than 200 Years and Counting. Crit. Care Clin..

[13] Akhtar T., Sheikh N., Abbasi M.H. (2014). Clinical and pathological features of Nerium oleander extract toxicosis in wistar rats. BMC Res. Notes.

[14] Kassop D., Donovan M., Cohee B.M., Mabe D.L., Wedam E.F., Atwood J.E. (2014). An unusual case of cardiac glycoside toxicity. Int. J. Cardiol..

[15] Richfield D. (2014). Medical gallery of Blausen Medical 2014. WikiJournal Med..

[16] Ni D., Madden T.L., Johansen M., Felix E., Ho D.H., Newman R.A. (2002). Murine pharmacokinetics and metabolism of oleandrin, a cytotoxic component of Nerium oleander. J. Exp. Ther. Oncol..

[17] Botha C.J., Penrith M.L. (2008). Poisonous plants of veterinary and human importance in southern Africa. J. Ethnopharmacol..

[18] Hugues T., Arnoult M., Beau N., Yaici K., Mélandri P., Saoudi N., Gibelin P. (2012). Intoxication volontaire au laurier rose; cas clinique et revue de la littérature. Ann. Cardiol. Angeiol..

[19] Turner J.L., Torres P. (2011). Oleander Poisoning of Horses. Guide B-712.

[20] Reiner A.C., Kass P.H., Magdesian K.G., Madigan J.E., Aleman M., Pusterla N. (2013). Oleander toxicosis in equids: 30 cases (1995–2010). J. Am. Vet. Med. Assoc..

[21] Butler J., Khan S., Scarzella G. (2016). Fatal Oleander Toxicosis in Two Miniature Horses. J. Am. Anim. Hosp. Assoc..

[22] Smith P.A., Aldridge B.M., Kittleson M.D. (2003). Oleander toxicosis in a donkey. J. Vet. Intern. Med..

[23] Galey F.D., Holstege D.M., Plumlee K.H., Tor E., Johnson B., Anderson M.L., Blanchard P.C., Brown F. (1996). Diagnosis of oleander poisoning in livestock. J. Vet. Diagn. Investig..

[24] McGuirk S.M., Semrad S.D. (2005). Toxicologic emergencies in cattle. Vet. Clin. Food Anim..

[25] Soto-Blanco B., Fontenele-Neto J.D., Silva D.M., Reis P.F., Nóbrega J.E. (2006). Acute cattle intoxication from Nerium oleander pods. Trop. Anim. Health Prod..

[26] Kozikowski T.A., Magdesian K.G., Puschner B. (2009). Oleander intoxication in New World camelids: 12 cases (1995–2006). J. Am. Vet. Med. Assoc..

[27] Van Saun R.J. (2009). Nutritional diseases of llamas and alpacas. Vet. Clin. Food Anim. Pract..

[28] Page C., Murtaugh R.J. (2015). Hypoglycemia associated with oleander toxicity in a dog. J. Med. Toxicol..

[29] Meyer H.P., Van Der Linden W.J., Van Der Linde-Sipman J.S. (1993). A case of oleander poisoning in a cat. Tijdschr. Diergeneeskd..

[30] LaBonde J. (1995). Toxicity in pet avian patients. Semin. Avian Exot. Pet Med..

[31] Martínez Monseny A., Martínez Sánchez L., Margarit Soler A., Trenchs Sainz de la Maza V., Luaces Cubells C. (2015). Poisonous plants: An ongoing problem. An. Pediatr..

[32] Wasfi I.A., Zorob O., Al katheeri N.A., Al Awadhi A.M. (2008). A fatal case of oleandrin poisoning. Forensic Sci. Int..

[33] Driggers D.A., Solbrig R., Steiner J.F., Swedberg J., Jewell G.S. (1989). Acute Oleander Poisoning. A Suicide Attempt in a Geriatric Patient. West. J. Med..

[34] Tracqui A., Kintz P., Branche F., Ludes B. (1998). Confirmation of oleander poisoning by HPLC/MS. Int. J. Legal Med..

[35] Gaillard Y., Pepin G. (1999). Poisoning by plant material: Review of human cases and analytical determination of main toxins by high-performance liquid chromatography–(tandem) mass spectrometry. J. Chromatogr. B Biomed. Sci. Appl..

[36] Bourgeois B., Incagnoli P., Hanna J., Tirard V. (2005). Traitement par anticorps antidigitalique d’une intoxication volontaire par laurier rose. Nerium oleander selfpoisoning treated with digoxin-specific antibodies. Ann. Fr. Anesth. Reanim..

[37] Hughes K.J., Dart A.J., Hodgson D.R. (2002). Suspected Nerium oleander (Oleander) poisoning in a horse. Aust. Vet. J..

[38] Radenkova-Saeva J., Atanasov P. (2013). Cardiac glycoside plants self-poisoning. Acta Med. Bulg..

[39] Kudo K., Tsuchihashi H., Ikeda N. (2003). Meeting challenges in forensic toxicology in Japan by liquid chromatography/mass spectrometry. Anal. Chim. Acta.

[40] Le Couteur D.G., Fisher A.A. (2002). Chronic and criminal administration of Nerium oleander. J. Toxicol. Clin. Toxicol..

[41] Rahnama-Moghadam S., Hillis L.D., Lange R.A. (2014). Environmental Toxins and the Heart. Heart and Toxins.

[42] Senthilkumaran S., Meenakshisundaram R., Michaels A.D., Thirumalaikolundusubramanian P. (2011). Electrocardiographic changes during inhalational oleander toxicity. J. Electrocardiol..

[43] Strano-Rossi S., Odoardi S., Castrignanò E., Serpelloni G., Chiarotti M. (2015). Liquid chromatography–high resolution mass spectrometry(LC–HRMS) determination of stimulants, anorectic drugs and phosphodiesterase 5 inhibitors (PDE5I) in food supplements. J. Pharm. Biomed. Anal..

[44] Oryan A., Maham M., Rezakhani A., Maleki M. (1996). Morphological studies on experimental oleander poisoning in cattle. J. Vet. Med. A..

[45] Gasparini G., Beretta C. (1984). Oleandro. Tossicologia Veterinaria.

